# Editorial: Innovative strategies for enhancing crop productivity and soil health using PGPB and nano-organics

**DOI:** 10.3389/fmicb.2025.1672604

**Published:** 2025-08-12

**Authors:** Angela Racioppo, Viviana Martins, Barbara Speranza, Marta Laranjo

**Affiliations:** ^1^Department of Agriculture, Food, Natural Resources and Engineering, University of Foggia, Foggia, Italy; ^2^Centre of Molecular and Environmental Biology, Department of Biology, University of Minho, Braga, Portugal; ^3^MED – Mediterranean Institute for Agriculture, Environment and Development, Évora, Portugal; ^4^Departamento de Medicina Veterinária, Escola de Ciências e Tecnologia, CHANGE – Global Change and Sustainability Institute, Universidade de Évora, Évora, Portugal

**Keywords:** bacteria, symbiosis, root, plant growth, environmental stress, beneficial interaction, microbiome, adaption

The two main challenges facing the agricultural sector nowadays are protecting the environment and increasing productivity. The growing demand for food, coupled with environmental concerns and limited natural resources, requires us to think about how to achieve high production targets without compromising sustainability. For this reason, agricultural strategies must focus on adopting more efficient, innovative and environmental friendly practices. Conventional agriculture, based on methods like chemical fertilizers, synthetic pesticides, and intensive monocultures aimed at maximizing yields, has raised significant concerns, fueling an ongoing debate over the long-term sustainability of such practices. The challenges posed by soil fertility degradation, chemical pollution and the impact on biodiversity are forcing farmers and society to reassess the balance between the immediate benefits of intensive production and the need to preserve natural resources for future generations. The latest research findings indicate that the future of agricultural productivity and environmental sustainability, as well as the intelligent exploitation of the biological resources present in soils and cultivated ecosystems, is contingent on the harnessing of the intricate interactions between plants, beneficial microorganisms and natural compounds.

This Research Topic brings together a collection of articles that advance and broaden current approaches to sustainable agriculture, proposing and characterizing beneficial microorganisms and biostimulants with potential to improve crop resilience and productivity by reducing dependence on chemical inputs.

One of the most interesting examples of this new frontier is the study of Tebele et al. on *Myrothamnus flabellifolia*, the so-called “resurrection plant,” famous for its amazing ability to survive long periods of extreme drought. The study analyses the changes occurring in the root microbiome during the dehydration and rehydration processes. Under conditions of severe water stress, *Actinomycetota* bacteria were predominant in the roots. These bacteria activate key genes involved in defense processes against oxidative stress and in synthesizing protective sugars, such as trehalose. As rehydration begins, the microbial community shifts in favor of *Pseudomonadota* bacteria, which facilitate the reconstruction of plant structures and the mobilization of nutrients. This intricate interaction between plants and microorganisms sheds new light on drought tolerance mechanisms and suggests ways to improve the resilience of food crops by targeting the root microbiome. The main finding is that the root microbiome acts as a multifunctional partner for plants, enhancing their capacity to adapt to abiotic and biotic stresses. There are effective methods to develop crops that are more resistant to the effects of climate change and environmental instability. These include modulating this alliance through agronomic practices, microbial inoculation or varietal selection.

The fight against plant diseases remains one of the most pressing challenges for global food security, and genomic microbiology provides new tools to understand the mechanisms and define innovative strategies. For instance, the genomic study by Liu et al. of *Neostagonosporella sichuanensis*, the causal agent of bamboo rhombic spot disease, sheds light on the ecological and economic importance of this species. Detailed genome analysis revealed a highly organized, bipartite structure with regions of repeated sequences that facilitate the pathogen's evolutionary adaptability. The high specialization of this fungus was found to derive from its ability to selectively degrade the host plant's cell wall.

Decoding the molecular mechanisms of aggression and identifying specific genetic profiles, such as those linked to pathogen effectors and degradative enzymes, opens new possibilities for developing more targeted and sustainable biocontrol strategies. This may reduce dependence on synthetic pesticides and protect higher-value crops, such as bamboo.

Another key pathway for the agroecological transition is the partial replacement of chemical fertilizers with beneficial soil microorganisms. The study by Sharma et al. highlights the potential of beneficial soil bacteria, such as *Pantoea agglomerans* strains. These microorganisms can solubilise phosphorus, enabling plants to absorb nutrients more efficiently. They also demonstrate remarkable resistance to salinity, oxidative stress, and metal contaminants. The inoculation of these bacteria onto wheat seeds significantly increased growth and yield, in terms of both the number of ears and grain weight. It also improved the structure of the root microbiome and promoted the presence of beneficial microbial groups throughout the growth cycle. Genomic analysis reveals that these strains lack virulence genes but possess genetic determinants associated with plant hormone production, integrated stress resistance, and strengthening crop symbioses. These results consolidate the prospect that biofertilizers can support productivity while preserving soil quality and integrity.

Finally, fresh perspectives for a circular bioeconomy are presented in the research of Zhou et al. on the utilization of wood vinegar as a natural biostimulant for tomato cultivation. Being a by-product of biomass pyrolysis, wood vinegar contains fractions of organic acids and phenolic compounds that stimulate plant development and soil fertility. The results demonstrate that individual treatments with these fractions significantly increase nutrient availability, beneficial microbial diversity, and the hormonal regulation of plant growth and resilience. However, combining the two types of compounds reduces these benefits by generating antagonistic effects.

This research valorises a renewable resource derived from the sustainable management of wood waste and highlights the need to develop refined formulations and targeted protocols for using natural inputs, thus avoiding indiscriminate applications that could compromise their effectiveness.

In summary, studies show that to move toward regenerative and resilient agriculture, we must firstly understand and manage microbiomes, enhance biological and natural inputs, and integrate basic science, agronomic innovation and policy in different sectors. Specific tools that can improve productivity, crop health and soil quality include the targeted introduction of beneficial microorganisms, the selection of resilient varieties, and the adoption of biostimulants derived from wastes or by-products. These measures can help to create an agricultural system that can meet future challenges without harming the environment.

Investing in the integration of microbiological research, sustainable agricultural practices and technological innovation is essential to build robust and productive agri-food systems that can safeguard natural resources for future generations.

